# Supplementary material for: Label-Free Mass Spectrometry-Based Quantification of Linker Histone H1 Variants in Clinical Samples
Source: Int J Mol Sci. 2020 Oct 4;21(19):7330. doi: 10.3390/ijms21197330 (PMC7582528; doi:10.3390/ijms21197330)
Supplement: Supplementary file 1 [file ijms-21-07330-s001.pdf]

Supplementary Tables

Table S1. Triple-Negative Breast Cancer Samples.

Sample	Relapse	Grade	Stage[1]		ER[2]	PR[3]	HER2[4]	HER2	Ki67	Tumor%
			pT	pN	%	%	Score	%	%	
**1233**	**NO**	**3**	**2**	x	**Neg**	**Neg**	**Neg**		40	50
1255	NO	3	1c	1a	Neg	Neg	Neg		70	60
1375	NO	3	2	0	Neg	Neg	Neg		65	60
1446	NO	3	2	1a	Neg	Neg	Neg		80	60
12110	NO	3	2	0(sn)	Neg	Neg	Neg		70	80
12132	NO	3	1c	0(sn)	Neg	Neg	Neg		40	40
12378	NO	3	1c	1mi	Neg	Neg	1+		60	60
12461	NO	3	2	0(sn)	Neg	Neg	Neg		70	60
13137	NO	3	2	1a	Neg	Neg	Neg		70	80
13188	NO	3	2(m)	3a	Neg	Neg	Neg		24	60
13382	NO	3	2	2a	Neg	Neg	Neg		70	50
13607	NO	3	2	1mi	Neg	Neg	1+	20	85	50
13662	NO	2	1c	x	Neg	Neg	Neg		28	60
14132	NO	3	1c	1a	Neg	Neg	Neg		45	70
14501	NO	3	2	0	Neg	Neg	Neg		75	70
14534	NO	3	1c	0(sn)	Neg	Neg	1+	30	40	65
PN11188	NO	3	2(m,is)	1a	Neg	Neg	Neg		70	70
PN1260	NO	3	2	1a	Neg	Neg	Neg		45	50
1427	YES	3	1c	0	Neg	Neg	2+	20	45	60
1516	YES	3	2(m)	2a	Neg	Neg	Neg		35	65
12116	YES	3	3	y1a	Neg	Neg	Neg		80	85
12118	YES		y2	3a	Neg	Neg	Neg		40	70
12233	YES	3	2(m)	1a	Neg	Neg	Neg		55	50
12362	YES	3	1c(is)	1a	Neg	Neg	Neg		80	40
12424	YES	3	2(m)	2a	Neg	Neg	1+	20	35	85
13211	YES	2	1c	0(sn)	Neg	Neg	Neg		80	50
13282	YES	2	3	2a	Neg	Neg	1+	30	15	40
13441	YES	3	2	1a	Neg	Neg	Neg		35	50
13452	YES	3	2	2a	Neg	Neg	1+	20	60	70
13693	YES	3	2	0(sn)	Neg	Neg	Neg		30	60
14125	YES	3	2(is)	3b	Neg	Neg	Neg		70	50
14498	YES	3	2	0(sn)	Neg	Neg	Neg		70	40
14580	YES	3	2	0(sn)	Neg	Neg	Neg		85	65
14754	YES		3	3a	Neg	Neg	1+	20	80	70
15625	YES	3	1c	3c	Neg	Neg	Neg		90	40
PN12193	YES	NA	y3	y3a	Neg	Neg	Neg		90	40
PN12383	YES	3	3(m)	3a	Neg	Neg	2+	20	40	70

[1] Based on the TNM staging system; [2] ER: estrogen receptor, [3] PR: progesteron receptor, [4] HER2: human epidermal growth factor receptor 2.

Table S2. LFQ Values for Histone H1 Variants in the Experiments Involving MEFs.

Sample			Histone H1.0	Histone H1.2	Histone H1.4	Histone H1.1	Histone H1.5	Histone H1.3	Histone H1x
Lysates		WT	3.07E+08	6.74E+09	7.79E+08	6.13E+08	1.48E+09	4.4E+08	39817000
			3.64E+08	7.09E+09	6.56E+08	6.71E+08	1.54E+09	3.38E+08	37475000
			3.08E+08	4.9E+09	4.76E+08	5.53E+08	1.06E+09	2.95E+08	42211000
		H1.0 KD	1.53E+08	1.3E+10	1.3E+09	1.44E+09	2.74E+09	9.08E+08	59576000
			1.33E+08	9.74E+09	9.6E+08	1.3E+09	2.12E+09	6.11E+08	42663000
			1.75E+08	1.5E+10	1.51E+09	1.71E+09	3.66E+09	1.05E+09	
Nuclei	3 g	WT	1.99E+09	3.87E+10	3.85E+09	3.21E+09	8.36E+09	1.77E+09	84987000
			2.67E+09	5.55E+10	5.23E+09	4.33E+09	1.31E+10	3.42E+09	1.62E+08
			2.14E+09	3.13E+10	2.22E+09	2.43E+09	4.82E+09	1.29E+09	1.52E+08
		H1.0 KD	6.61E+08	8.02E+10	7.33E+09	7.72E+09	1.8E+10	5.55E+09	1.52E+08
			5.45E+08	6.44E+10	5.91E+09	6.25E+09	1.33E+10	4.11E+09	1.07E+08
			6.57E+08	7.84E+10	6.77E+09	7.13E+09	1.66E+10	4.72E+09	1.24E+08
	1:5 dilution	WT	1.96E+09	3.88E+10	3.48E+09	2.98E+09	7.05E+09	2.1E+09	69892000
			2.69E+09	7.2E+10	6.04E+09	5.04E+09	1.33E+10	3.96E+09	1.19E+08
			2.13E+09	3.24E+10	2.21E+09	2.54E+09	5.18E+09	1.47E+09	77838000
		H1.0 KD	7.75E+08	1.07E+11	8.39E+09	9.05E+09	1.74E+10	8.95E+09	1.27E+08
			4.94E+08	7.62E+10	5.89E+09	6.36E+09	1.29E+10	4.63E+09	1.46E+08
			6.24E+08	9.72E+10	8.3E+09	7.95E+09	1.72E+10	5.63E+09	1.4E+08
	1:25 dilution	WT	2.03E+09	4.54E+10	3.75E+09	3.42E+09	7.48E+09	2.47E+09	1.15E+08
			2.41E+09	6.6E+10	5.12E+09	4.62E+09	1.09E+10	4.32E+09	55540000
			2.11E+09	3.09E+10	2.06E+09	2.15E+09	4.77E+09	1.88E+09	
		H1.0 KD	9.54E+08	1.25E+11	9.83E+09	1.01E+10	2.07E+10	5.9E+09	1.06E+08
			7.8E+08	1.07E+11	7.63E+09	8.02E+09	1.7E+10	4.83E+09	1.09E+08
			9.11E+08	1.17E+11	9.77E+09	9.36E+09	2.14E+10	5.5E+09	68468000

1:125 dilution	WT	3.55E+09	6.22E+10	5.36E+09	4.24E+09	9.18E+09	3.38E+09	62184000
		5.61E+09	5.56E+10	2.22E+09	3.69E+09	8.74E+09	3.1E+09	
		3.11E+09	1.4E+10	4.81E+08	1.6E+09	1.91E+09	3.19E+09	
	H1.0 KD	5.89E+08	4.42E+10	1.48E+09	4.75E+09	5.46E+09	1.53E+09	
		9.43E+08	5.04E+10	1.98E+09	4.67E+09	8.47E+09	5.45E+09	
		5.94E+08	2.67E+10	8.63E+08	3.7E+09	5.26E+09	4.57E+09	

Table S3. LFQ Values for Histone H1 Variants in the Experiments Involving Mouse Pancreas.

Sample		Histone H1.0	Histone H1.2	Histone H1.4	Histone H1.1	Histone H1.5	Histone H1.3
FFPE	slice_1	3.11E+07	1.05E+08	2.61E+07	1.19E+06	1.65E+07	1.78E+06
	slice_2	2.84E+07	2.09E+08	2.43E+07	3.40E+06	2.79E+07	3.08E+06
	20000	4.48E+07	9.25E+08	5.37E+07	7.89E+05	3.47E+08	2.24E+06
	5000	4.84E+07	3.43E+09	1.25E+08	1.18E+06	9.97E+08	3.23E+06
	2500	5.07E+07	3.94E+09	1.55E+08	0.00E+00	1.70E+09	4.79E+06
	1000	8.80E+07	5.34E+09	2.18E+08	0.00E+00	1.86E+09	2.74E+06
OCT	slice_1	5.76E+07	3.37E+08	5.33E+07	9.80E+06	3.95E+07	4.08E+06
	slice_2	4.75E+07	1.90E+08	4.00E+07	4.15E+06	2.58E+07	1.88E+06
	20000_1	7.57E+07	7.23E+08	7.00E+07	1.16E+06	2.16E+08	2.16E+06
	20000_2	8.15E+07	6.25E+08	6.38E+07	1.69E+06	1.70E+08	4.09E+06
	5000_1	7.60E+07	2.42E+09	1.12E+08	1.24E+06	6.32E+08	4.41E+06
	5000_2	6.79E+07	1.39E+09	7.96E+07	2.08E+06	4.03E+08	3.11E+06
	2500_1	8.76E+07	2.99E+09	1.92E+08	2.94E+06	8.59E+08	6.08E+06
	2500_2	5.94E+07	1.97E+09	1.17E+08	1.11E+06	6.50E+08	1.76E+06
	1000_1	5.34E+07	2.36E+09	2.09E+08	2.00E+06	8.19E+08	1.35E+06
	1000_2	1.13E+08	4.16E+09	2.66E+08	0.00E+00	1.60E+09	2.40E+06

Table S4. LFQ Values for Histone H1 Variants in the Experiments Involving Laser Micro-dissected Breast Cancer Samples.

Sample		Histone H1.0	Histone H1.5	Histone H1.2	Histone H1.1	Histone H1x
Normal	1	1.7E+08	1.35E+08	4.28E+08	2485200	35356000
	2	1.03E+08	3.08E+08	6.46E+08	4790400	17827000
	3	1.92E+08	3.47E+08	7.77E+08	4161200	16014000
	4	1.09E+08	1.82E+08	4.86E+08	0	10396000
Tumor 1	1	81409000	1.23E+08	3.05E+08	3089600	7104500
	2	1.85E+08	3.51E+08	9.62E+08	4327100	22729000
	3	72981000	71598000	2.67E+08	1906600	6260500
	4	1.08E+08	2.36E+08	4.92E+08	5009300	10171000
Tumor 2	1	1.7E+08	3.32E+08	7.64E+08	5855900	21465000
	2	83466000	85949000	2.86E+08	3389800	10194000
	3	48388000	91149000	2.62E+08	2611200	9769600
	4	45131000	62583000	2.15E+08	1544200	5633100

Table S5. LFQ Values for Histone H1 Variants in Triple-Negative Breast Cancer Samples (Dataset 1).

Sample	Condition	Histone H1.0	Histone H1.4	Histone H1.5	Histone H1.3	Histone H1.2	Histone H1.1	Histone H1x
**12110**	no relapse	5.05E+08	1.62E+07	1.30E+09	1.03E+08	3.94E+09	1.95E+08	1.16E+08
**12132**	no relapse	1.00E+09	2.12E+07	2.02E+09	9.91E+07	6.59E+09	2.76E+07	1.86E+08
**1233**	no relapse	9.30E+08	5.75E+07	1.67E+09	3.49E+07	6.63E+09	6.82E+07	3.01E+08
**12378**	no relapse	7.18E+08	2.95E+07	9.90E+08	1.23E+08	4.00E+09	3.55E+07	2.05E+08
**12461**	no relapse	1.73E+08	1.54E+07	7.52E+08	5.85E+07	2.12E+09	3.57E+07	1.22E+08
**1255**	no relapse	6.88E+08	2.25E+07	2.34E+09	1.00E+08	3.93E+09	6.91E+07	2.18E+08
**13133**	no relapse	3.09E+09	4.47E+07	3.29E+09	2.35E+08	7.84E+09	7.28E+07	4.15E+08
**13137**	no relapse	2.60E+09	2.35E+07	2.83E+09	1.88E+08	7.82E+09	6.88E+07	3.24E+08
**13188**	no relapse	1.60E+09	1.67E+07	1.92E+09	2.17E+08	7.51E+09	1.14E+08	3.62E+08
**13382**	no relapse	8.83E+08	4.27E+07	1.69E+09	1.02E+08	5.11E+09	3.95E+07	2.26E+08
**13607**	no relapse	8.21E+08	5.79E+07	1.59E+09	2.51E+08	7.75E+09	4.57E+07	2.59E+08
**13662**	no relapse	9.21E+08	2.55E+07	9.32E+08	2.56E+07	3.03E+09	3.92E+07	1.44E+08
**1375**	no relapse	1.51E+09	2.01E+07	1.44E+09	8.69E+07	4.27E+09	5.69E+07	2.36E+08
**14132**	no relapse	1.10E+09	2.31E+07	1.02E+09	8.14E+07	5.53E+09	2.58E+07	1.76E+08
**1446**	no relapse	1.80E+09	5.33E+07	1.17E+09	2.57E+08	6.07E+09	3.39E+07	4.81E+08
**14501**	no relapse	2.31E+08		1.45E+08	1.44E+07	4.97E+08	9.83E+06	8.88E+07
**14534**	no relapse	5.93E+08		7.12E+08	4.05E+07	2.02E+09	7.49E+06	8.95E+07
**PN11188**	no relapse	6.64E+08	1.77E+07	5.97E+08	6.95E+07	2.48E+09	4.72E+07	1.45E+08
**PN1260**	no relapse	5.75E+08	1.83E+07	5.03E+08	6.78E+07	2.55E+09	4.56E+07	2.08E+08
**12116**	relapse	4.25E+08	2.90E+07	2.09E+09	1.46E+08	5.33E+09	1.60E+07	1.89E+08
**12188**	relapse	7.12E+08	1.34E+07	6.52E+08	5.53E+07	9.22E+08	3.03E+07	1.77E+08
**12233**	relapse	2.14E+08	1.48E+07	2.13E+08	1.26E+07	7.12E+08	7.30E+06	4.59E+07
**12362**	relapse	5.57E+08	2.12E+07	6.37E+08	2.65E+07	1.97E+09	2.76E+07	5.83E+07
**12424**	relapse	3.48E+08	1.40E+07	1.21E+09	9.14E+07	3.61E+09	2.86E+07	1.87E+08
**13211**	relapse	3.12E+08		9.94E+08	5.15E+07	2.70E+09	3.93E+07	6.23E+07
**13282**	relapse	7.23E+08		1.76E+09	2.64E+08	8.19E+09	3.04E+07	3.20E+08
**13452**	relapse	1.50E+09		1.51E+09	8.77E+07	4.65E+09	3.48E+07	2.14E+08
**13693**	relapse	8.45E+07		2.80E+08	1.15E+07	5.75E+08	2.29E+07	7.02E+07
**14125**	relapse	6.69E+08	1.55E+07	7.52E+08	4.89E+07	2.54E+09	2.01E+07	1.94E+08
**1427**	relapse	9.90E+07		1.74E+08	1.42E+07	5.71E+08	7.36E+06	6.68E+07
**14498**	relapse	1.25E+09		2.06E+09	1.11E+08	5.41E+09	1.54E+08	1.07E+08
**14580**	relapse	6.13E+08	7.09E+06	1.33E+09	7.04E+07	3.89E+09	1.40E+07	7.89E+07
**14784**	relapse	3.60E+08		5.89E+07	6.21E+06	1.92E+08	3.81E+06	1.16E+08
**1516**	relapse	9.31E+08	1.82E+07	7.16E+08	6.89E+07	2.65E+09	5.06E+07	1.76E+08
**15625**	relapse	2.51E+08	1.39E+07	1.84E+08	3.07E+07	8.47E+08	4.64E+06	9.73E+07
**PN12193**	relapse	7.47E+07		3.93E+08	1.65E+07	8.12E+08	1.04E+07	9.09E+07
**PN12383**	relapse	4.06E+08		1.61E+08	1.44E+07	5.24E+08	3.82E+06	1.23E+08

Table S6. LFQ Values for Histone H1 Variants in Triple-Negative Breast Cancer Samples (Dataset 2).

Sample	Condition	Histone H1.0	Histone H1.5	Histone H1.3	Histone H1.2	Histone H1x
**1**	no relapse	1.16E+08	2.69E+08	78261000	6.41E+08	65420000
**2**	no relapse	1.33E+08	2.62E+08	19672000	1.07E+09	47054000
**3**	no relapse	1.54E+08	1.87E+08	13668000	6.81E+08	43573000
**4**	no relapse	1.54E+08	2.51E+08	23580000	1.2E+09	39388000
**5**	no relapse	73861000	3.55E+08	36834000	1.28E+09	48367000

**6**	relapse	2.42E+08	1.34E+08	7819400	6.28E+08	15683000
**7**	relapse	2.77E+08	86175000	10231000	4.95E+08	24810000
**8**	relapse	1.76E+08	2.63E+08	14627000	8.62E+08	18922000
**9**	relapse	90996000	1.21E+08	7598300	3.64E+08	24274000

Supplementary Figures

A

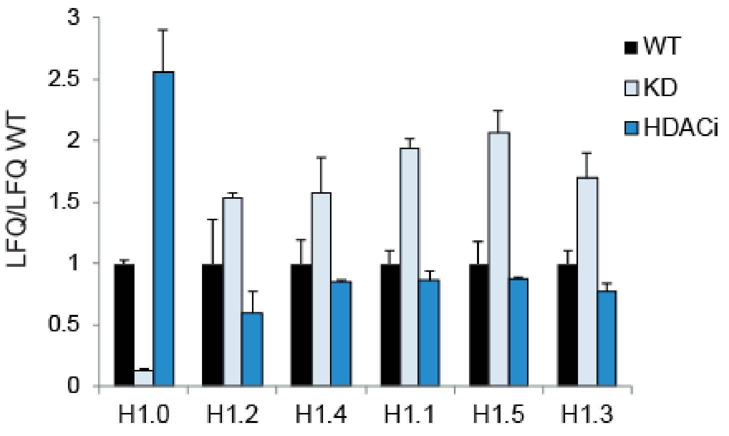

B

Histone	KD	KD:HDACi =2:1	KD:HDACi =1:1	KD:HDACi =1:2	KD:HDACi =1:3	HDACi	
**H1.0**	9.29E+07	1.91E+08	2.21E+08	3.11E+08	4.11E+08	6.70E+08	**LFQ values**
**H1.2**	1.10E+10	1.22E+10	9.18E+09	8.02E+09	6.76E+09	3.61E+09	
**H1.4**	6.53E+08	6.07E+08	5.51E+08	5.71E+08	3.76E+08	1.88E+08	
**H1.1**	9.94E+08	9.30E+08	7.39E+08	7.17E+08	5.08E+08	2.90E+08	
**H1.5**	1.88E+09	1.70E+09	1.86E+09	1.54E+09	9.87E+08	6.20E+08	
**H1.3**	3.83E+08	3.65E+08	2.91E+08	1.03E+08	2.43E+08	6.27E+07	
**H1.0**	**1.00**	2.06	2.38	3.35	4.42	**7.21**	**Observed fold change (LFQ values normalized over KD)**
**H1.2**	**1.00**	1.11	0.83	0.73	0.61	**0.33**	
**H1.4**	**1.00**	0.93	0.84	0.87	0.58	**0.29**	
**H1.1**	**1.00**	0.94	0.74	0.72	0.51	**0.29**	
**H1.5**	**1.00**	0.90	0.99	0.82	0.53	**0.33**	
**H1.3**	**1.00**	0.95	0.76	0.27	0.63	**0.16**	
**H1.0**	**1.00**	3.04	4.10	5.09	5.65	**7.21**	**Expected fold change**
**H1.2**	**1.00**	0.77	0.66	0.55	0.50	**0.33**	
**H1.4**	**1.00**	0.76	0.64	0.52	0.47	**0.29**	
**H1.1**	**1.00**	0.76	0.65	0.52	0.47	**0.29**	
**H1.5**	**1.00**	0.77	0.67	0.55	0.50	**0.33**	
**H1.3**	**1.00**	0.71	0.58	0.44	0.37	**0.16**	

Figure S1. Linearity assessment. (**A**) Histone H1 protein levels in MEF cells WT, H1.0 KD and treated with the HDAC inhibitor (HDACi), normalized over the WT condition. Error bars represent the SEM from biological triplicates. (**B**) LFQ values, observed fold changes and expected fold changes for histone H1 isoforms quantified from samples deriving from mixing HDACi treated cells and KD cells in different proportions. The observed fold changes were calculated by dividing the LFQ values for the different samples by the LFQ value for the KD sample. The expected fold changes for the KD and the HDACi samples were the same as the observed fold changes. The expected fold changes for the other points were calculated based on the different proportion of HDACi and KD present in the sample.

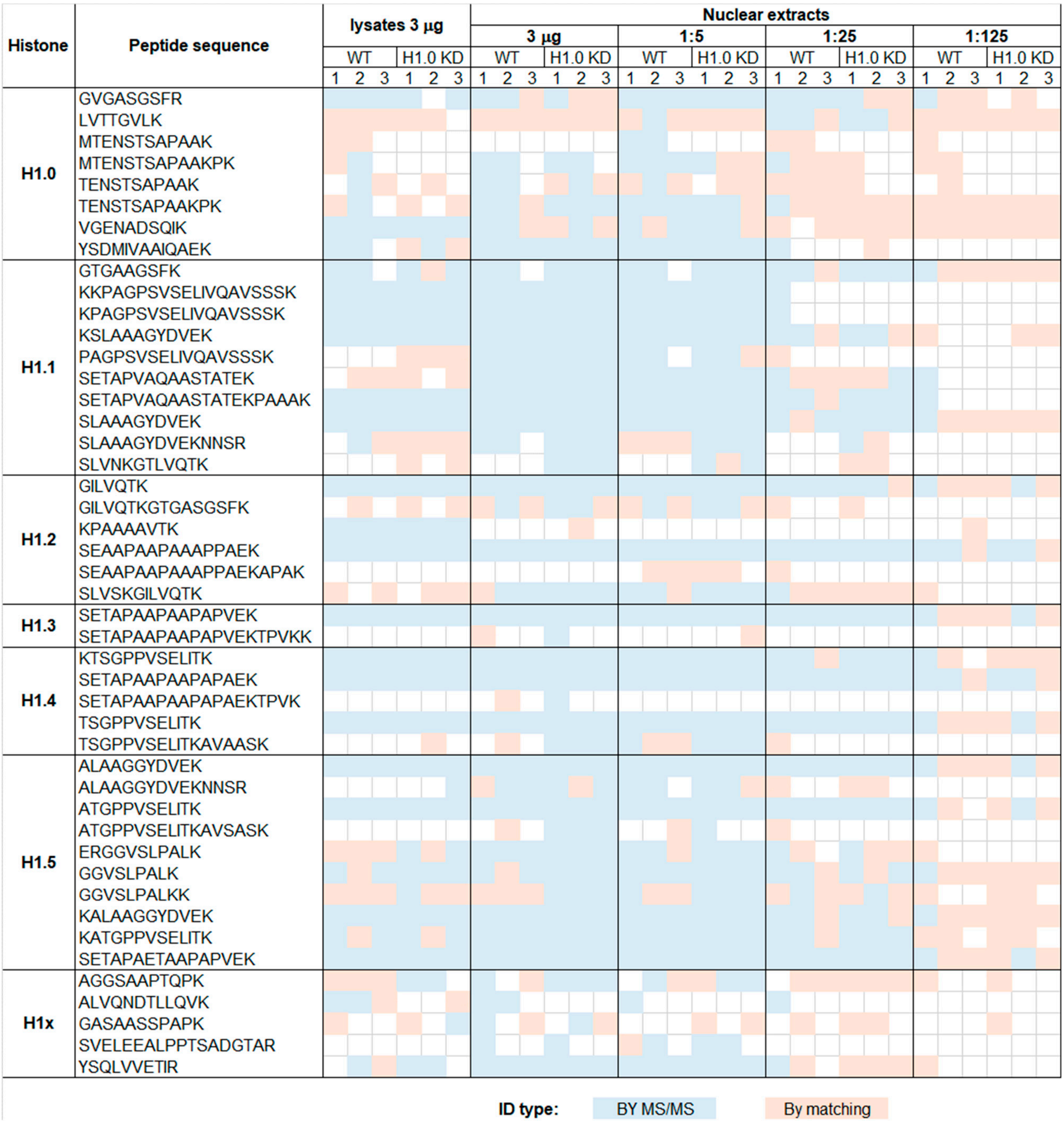

Figure S2. Unique peptides quantified from serial dilutions of MEF extracts. Unique peptides with up to two miscleavages identified from the samples described in Figures 1 and 3.

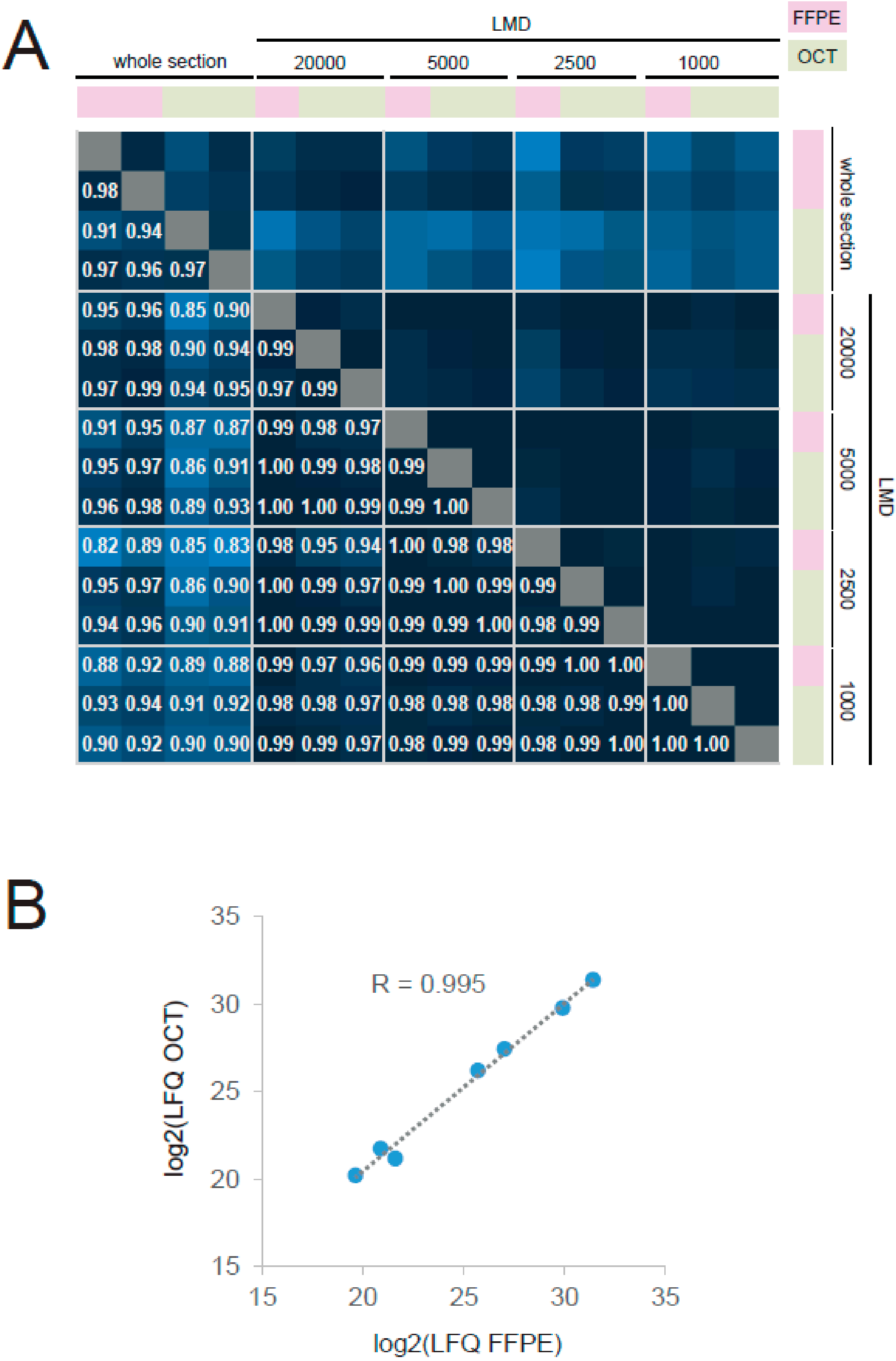

Figure S3. Quantification of histone H1 variants from laser microdissected pancreas samples. (**A**) Correlation matrix based on Pearson correlation coefficients of log2 MaxLFQ values for histone H1 variant in the whole sections and LMD samples shown in Figure 4. (**B**) Correlation of the log2(MaxLFQ) values for OCT and FFPE samples (20,000 laser microdissected cells). R: Pearson's correlation score.

A

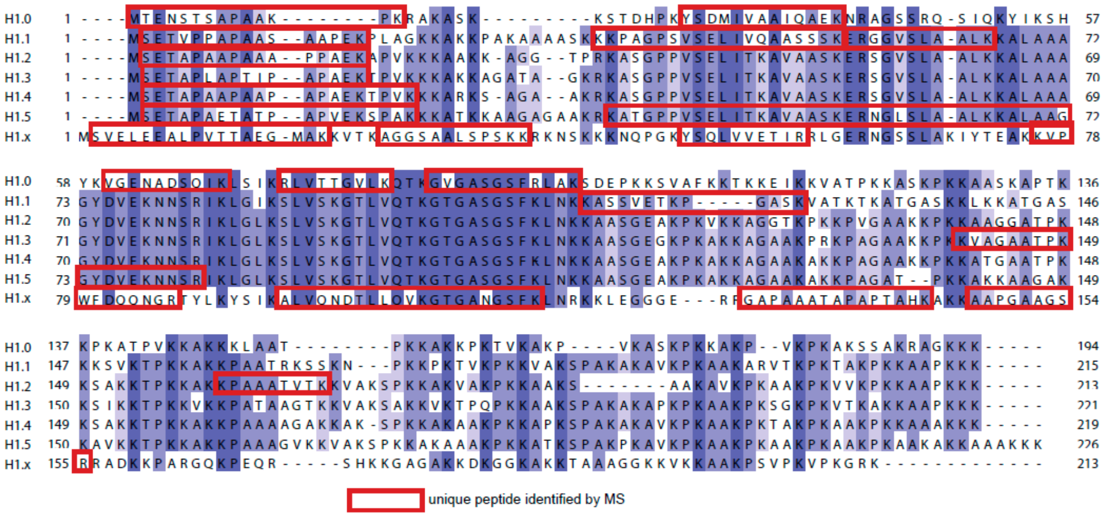

B

Histone	Sequence	Sample																																			
		1	2	3	4	5	6	7	8	9	10	11	12	13	14	15	16	17	18	19	20	21	22	23	24	25	26	27	28	29	30	31	32	33	34	35	36	37	38
H1.0	GVGASGSFR	1	1	1	1	1	1	1	1	1	1	1	1	1	1	1	1	2	1	1	1	1	1	1	1	1	1	1	1	1	1	1	1	1	2	1	1	1	1
	LVTTGVLK	1	1	2	1	1	1	1	1	1	1	1	1	2	1	1	1	1	1	1	1	1	1	1	1	1	1	1	2	1	1	2	1	1	1	1	1	1	1
	MTENSTSAPAAK	1	1	1			1	1	1	1	1	1	1	1	1	1	1	1	1	1	1	1		1	1			1	1	1	1		1	1	1			1	1
	MTENSTSAPAAKPK	1	1	1	1	1	1	1	2	1	1	1	2	2	1	1		2	1	2	1	1	1	1	1	1	1	1	2	1	1	1	1	1	1	1	1		1
	RLVTTGVLK	1		1	1										1	1		1	1					1	1	1	1	1	1		1		1	1			1	1	1
	TENSTSAPAAK	3	4	2	2	3	3	2	4	3	2	3	3	3	3	2	3	3	3	3	3	4	1	3	3	3	1	4	3	3	2	2	4	3	3	3	1	3	3
	TENSTSAPAAKPK	5	5	3	2	4	5	4	5	4	3	6	6	4	5	4	5	6	3	3	3	6	2	5	5	4	2	7	7	5	4	4	3	5	3	5	2	5	7
	VGENADSQIK	1	1	3	1	1	2	2	2	1	1	2	2	2	3	1	1	2	1	3	1	2	1	2	1	1	1	3	1	2	1	1	1	2	1	2	1	1	2
	YSDMIVAAIQAEK	5	5	8	7	6	4	5	7	4	4	8	12	6	9	5	7	6	6	7	9	4	2	8	7	8	5	7	7	6	6	7	6	8	6	7	3	8	6
H1.1	ASSVETKPGASK	3	2	2	2	2	2	2	2	2	2	2	3	2	2	2	2	2		2	2	2	2	2	2	1	2	2	2	2	1	1	1	2	2	2	2	2	2
	ERGGVSLAALK	1	1	1		1	1	1	1	1	1	1	1	1	1	1	1	1			1	1	1	1	1	1		1	1	1	1	1		1		1	1		1
	GGVSLAALK	1		2			1			1			1	1	1		2				1			1		1			1		1	1		1					2
	GGVSLAALKK	1	1	1	1	1	1	1	1	1	1	1	1	1	1	1	1	1		1		1	1	1					1	1	1	1		1		1	1		
	KASSVETKPGASK	1	1	1		1			1			1		1				1			1				1		1	1	1	1	1		1				1		
	KKPAGPSVSELIVQAASSSK	2	1	2	2	1	2		2	2	2	2	2	2	2	2	1	2		2	2	2	1	1	2	1	2	2	2	2	2	1		2	2	2	2	1	2
	KPAGPSVSELIVQAASSSK	1		1	1		1			1	1	1	1	1	1	1	1	1		1	1	1	1	1	1	1		1	1	1	1	1	1			1			1
	SETVPPAPAASAAPEKPLAGK	2			2		2		1	1	2	2	2	1	2	1					1	1	1	2					2					1		1	1		1
H1.2	KPAAATVTK	1	1	1	1	1	1	1	1	1	1	1	1	1	1	1	1	1	1	1	1	1	1	1	1	1	1	1	1	1	1	1		1	1	1	1	1	1
	PAAATVTK					1								1	1			1	1	1	1							1											1
	PAAATVTKK														1											1							1						
	SETAPAAPAAAPPAEK	6	6	6	4	4	6	4	5	5	7	7	6	7	6	9	6	6	5	8	5	6	6	6	5	5	6	7	6	3	8	6	3	5	5	4	5	3	4
	SETAPAAPAAAPPAEKAPVK	1	2	1	2		2		2	2	1	1	1	2	1	1	1	2		2	2			2	2	2		1	1			1		1		1			
H1.3	KVAGAATPK	2	1	1	1	1	2	1	1	2	1	1	1	1	1	1	1	2		1	1	1	1	1	1	1	1	2	1	1	2	2	1	1	1	1	1	1	2
	SETAPLAPTIPAPAEK	3	4	4	3	5	2	1	3	4	4	5	4	3	4	4	5	3	2	4	3	2	1	3	4	3	2	5	3	1	2	4	1	4	4	4	1	1	4
H1.4	SETAPAAPAAPAPAEK	1	3	1	2	2	4	2	2	2	1	2	4	1	2	2		2	1		1	2		1	1	2	1	2	2	1	1	2	1	2	3	2	1		3
	SETAPAAPAAPAPAEKTPVK	1	1	1	1		1		1	1	1	1	1	1	1		1	1			1	1		1	1	1		1				1		1		1			1
H1.5	ALAAGGYDVEK	3	2	3	3	3	3	3	2	3	1	2	1	1	3	3	1	3	1	1	3	1	2	3	2	1	2	2	1	1	2	3	2	2	2	2	2	1	1
	ALAAGGYDVEKNNSR	2	1	2			1		2	1		1	2	2		1	2	1		2	1		1	2	1		1	2	2		2	2		1		1	1		1
	ATGPPVSELITK	1	1	1	1	1	1	2	1	1	1	1	1	1	1	1	1	1		1	1	1	1	1	2	2	1	1	1	1	1	1	1	2	1	1	1	1	1
	ATGPPVSELITKAVAASK	1	1	1	1		1		1	1	1	1	1	1	1	1	1	1		1	1	1	1	1	1	1		1	1	1	1	1		1	1	1	1	1	1
	ERNGLSLAALK	2	2	2	2	1	2	2	1	2	2	2	2	2	2	2	2	2		2	1	1	2	2	2	1		2	2	2	2	2		1	1	1	1		2
	ERNGLSLAALKK	1	1	1			1		1	1	1	1	1	1	1	1		1		1		1	1	1					1		1	1							
	KALAAGGYDVEK	2	3	2	2	2	2	2	2	2	2	2	3	3	4	2	2	3	1	2	2	2	2	4	2	2	2	2	2	2	2	1	2	2	2	2	2	2	2
	KATGPPVSELITK	3	3	2	4	3	4	3	4	3	2	3	3	4	4	3	3	2	1	3	3	2	2	3	2	2	3	2	3	2	2	3	1	3	2	4	2	2	3
	NGLSLAALK	2	2	2	2	2	2	2	2	2	2	2	2	2	2	2	2	2	2	2	2	2	2	2	2	2	2	2	2	2	2	2	2	2	2	2	2	2	2
	NGLSLAALKK	1	2	1	1	1	2	1	1	1	1	1	2	2	1	1	1	1	1	1	1	1	1	2	1	1	1	1	2	1	1	2		1	1	1	1	1	1
	SETAPAETATPAPVEK	7	7	7	5	5	7	6	6	7	5	7	7	6	6	6	6	6	4	7	7	6	4	6	6	6	3	7	5	4	7	6	2	5	6	6	5	4	6
	SETAPAETATPAPVEKSPAK		1	1	1				1	1	1	1	1	1	1		1	1			1	1		1					1			1	1			1			
H1x	AAPGAAGSR	2	2	1		2	1		2	1	1	2	1	1	3	1	1	2	1	2	1	2	1	1	1	1	1	1	2	1	2	1	2	2	1	2	2	2	2
	AGGSAALSPSK	2	2	1	2	2	2	3	2	3	1	2	1	2	1	1	2	2	1	1	1	1	1	2	1	2	3	1	3	2	2	1	2	1	2	1		3	2
	AGGSAALSPSKK				1		1			1	1	1	1	1	1		1	1				1	1	1	1	1	1	1		1	1					1			1
	ALVQNDTLLQVK	2	2	2	2	1	2	1	2	2	2	2	2	1	2	2	1	2	2	2	2	2	1	2	2	2	1	2	1	2	2	1	2	2	2	2	2	2	2
	GAPAAATAPAPTAHK	2	2	2	2	2	2	2	2	2	2	2	2	2	2	2	2	2	2	2	2	2	2	2	2	2	2	2	2	2	2	2	2	2	2	2	2	2	3
	GTGANGSFK	2	2	2	2	2	2	2	2	2	2	2	2	2	2	1	2	2	1	2	2	2	2	2	2	2		2	2	2	2	2	2	2	2	2	1	1	2
	KVPWFDQQNGR	2	2	1			1		2	2	2	2	1	2	2	1	2	2		2	2	1	1	2	2		1	2	1	2	1	1	2	2	2	2	2	2	2
	SVELEEALPVTTAEGMAK	5	4	2	4	2	3	4	4	4	3	4	3	4	3	3	4	4	2	4	3	3	3	4	3	3	2	5	3	3	2	1	3	3	2	3	2	4	3
	VPWFDQQNGR	2	2	1		1	1		1	1	1	1	1	1	1		1	1		2	1	1		1	1	1	1	1		1			1	1	1	1	1		1
	YSQLVVETIR	1	1	1	1	1	1	1	1	1	1	1	1	1	1	1	1	1	1	1	1	1	1	1	1	1	1	1	1	1	1	1	1	1	1	1	1	1	1
	YSQLVVETIRR	1	1	2	1		2	1	2		1							1		2	1		1	2				1	2	1	2	2		2	1				

Figure S4. MS analysis of human histone H1 variants. (**A**) Sequence alignment of human histone H1 variants. Unique sequences detected by MS are marked by red squares. (**B**) List of unique peptides with up to two miscleavages identified from the Triple Negative breast cancer samples described in Figure 5C-F. Peptides identified in less than three samples are not displayed. The number of peptides detected in each sample (including multiple identifications of peptides with different charges and with/without methionine oxidation or N-termina acetylation) is shown.
